# The Experience of International Students and Institutional Recommendations: A Comparison Between the Students From the Developing and Developed Regions

**DOI:** 10.3389/fpsyg.2021.667230

**Published:** 2021-08-13

**Authors:** Talat Qadeer, Muhammad Kashif Javed, Aqsa Manzoor, Min Wu, Syed Imran Zaman

**Affiliations:** ^1^Sichuan University, Chengdu, China; ^2^Department of Economics, Sapienza University of Rome, Rome, Italy; ^3^Jinnah University for Women, Karachi, Pakistan

**Keywords:** international student experience, students from developing regions, students from developed regions, student satisfaction, institutional recommendation

## Abstract

Prior studies on the experiences of international students in China have mostly focused on their academic, sociocultural, and accommodation experiences. Hence, student health and safety, discrimination, and the services by the International Student Office (ISO) have remained unexplored. Moreover, due to the motivational differences between the students from developing and developed regions, a study that samples students from both regions may depict an exact picture of the experience of international students. Therefore, the objective of this study is to examine the influence of the dimensions (including those dimensions that have been ignored) of the experience of international students on their satisfaction. In addition, we make recommendations regarding Chinese institutes for future students based on a comparison between the students from developing and developed regions. Using hierarchical regression analysis, this study reveals that educational and non-educational experiences vary among students from different regions. Therefore, based on developing (e.g., Asia and Africa) and developed (e.g., America, Europe, and Australia) regions, important recommendations are discussed regarding how educational institutions and the Chinese government could best allocate resources and introduce policies to improve the experience of international students.

## Introduction

International students strongly benefit both the host countries and the academic institutions (Beine et al., 2014). These students can drive campus internationalization and financial benefits for host nations, and they are essential to the prestige of institutions, general reputation, and cultural enhancement (Forbes-Mewett, 2016). If international students remain in the host nation after graduation, their expertise plays an important role in the growth of a competent workforce that lifts the development of the host nation (Beine et al., 2014). Moreover, international students often become excellent ambassadors of the host countries if they return to their home countries (Pandit, 2007).

Thus, these benefits result in increased international student searches for higher education around the world (Mok et al., 2021). Accordingly, the USA, the UK, and Australia fascinate the majority of overseas students from across the globe (Ammigan and Jones, 2018). Simultaneously, China is among those countries that have initiated policies to bolster the enrollment of international students and to retain them in the local labor markets. In this regard, the Chinese government aspires to host 500,000 students from around the world by 2020 (Institute of International Education, 2018).

To attract more international students, the government suggested several strategies such as offering more scholarships and programs with instruction in English (Ahmad and Shah, 2018). As a result of these policies, ~440,000 international students from 205 countries were studying at Chinese universities in 2016. Of these students, 49,022 international students from 183 countries were on scholarships (Wang and Byram, 2019). These statistics show that international students in China have also started to contribute to the Chinese economy.

Nevertheless, increasing numbers of international students do not necessarily imply that these students are content with their academic and non-academic experiences, nor do they imply whether these students are eager to endorse China as a terminus to other international students. Besides, a recent study revealed that as the number of international students increases in China, their emotional problems are also rising (Li et al., 2021). Researchers have recommended that given the economic and global importance of the international students, their experience should be considered as an issue of customer satisfaction (Wearring et al., 2015). In a study, Wekullo (2019) also recommended that future research should investigate the ways that international students react to their particular experiences. The experiences of international students often include the challenges of functioning in a different educational system and cultural setting at a great distance from their families and current social support links (Horne et al., 2018). However, because they compose an increasing and diverse population, the unique experiences of international students have traditionally been overlooked (Wekullo, 2019). The current literature lacks sufficient research to provide a comprehensive understanding of the expectations, motivations, and experiences of international students in non-native English-speaking countries (Calikoglu, 2018).

During a program of study, numerous factors can directly influence the experiences of foreign students abroad (Ammigan and Jones, 2018). These factors include the satisfaction of international students with academics, security, community engagement, relationships, and home life (Arambewela and Hall, 2013). Other important factors include academic, social, and perceived discrimination (Wekullo, 2019), and support services (Ammigan and Jones, 2018). Understanding the implications of these factors can help attract more foreign students by boosting the image of the University in other countries (Chelliah et al., 2019).

However, prior research on the international students who study in China focuses largely on their experience regarding learning (Ahmad and Shah, 2018; Wen et al., 2018; Fan et al., 2019; Wang and Byram, 2019), accommodations (Ding, 2016), and cultural settings (Yu, 2010; Tian and Lowe, 2014; Ping et al., 2019; Wang and Lin, 2019). The roles of the experiences of overseas students with support services, health, and safety services, and perceived discrimination are missing in the current literature. With this study, we hope to fill this void.

In the context of overseas students in China, Ahmad and Shah (2018) suggested that students from Asia and other developing countries may consider lower living costs or the availability of scholarships as some of the most significant factors when deciding to study in China. Therefore, it is essential to examine if there are any differences between students from developing and developed regions (Ahmad and Shah, 2018). We believe that the research addresses the concerns of previous studies through a comparison analysis between students from developed regions (e.g., Europe, America, and Australia) and those from developing regions (e.g., Asia and Africa). This research will develop insights into the experiences of international students by analyzing the related influences of the experiences of foreign students in China using their institutional recommendations to others and analyzing their attitudinal feedback. With a greater understanding of how overseas students share their personal experiences in China with future students, the government and Chinese universities can better adjust their strategies for managing foreign students to enjoy the full benefits of international students.

## Literature Review and Conceptual Framework

Past research has promoted the view of students as customers and of education as a service (Halbesleben et al., 2003). Therefore, to identify the dimensions (accommodation, academic, sociocultural, discrimination, health and safety, and support services) of service quality, assess the overall satisfaction of students, define the influence of the overall satisfaction of students on future recommendations, and the expectancy-disconfirmation paradigm (Oliver, 1980) along with the Service Quality (SERVQUAL) model is adopted as a basic theoretical framework for this study.

Customer satisfaction is related to the direction and size of disconfirmation, which is defined as the difference between the pre-choice expectations of an individual and the post-choice performance of the service as perceived by the customer (Oliver, 1980). The customer is satisfied if expectations are met or exceeded. When perceived performance falls below expectations, the customer is dissatisfied. However, the construct of service quality is defined as the judgment of a customer about the overall superiority or excellence of an entity. According to Arambewela and Hall (2009), SERVQUAL as an instrument has been widely used for assessing customer perceptions of service quality in service organizations.

**Figure 1:**
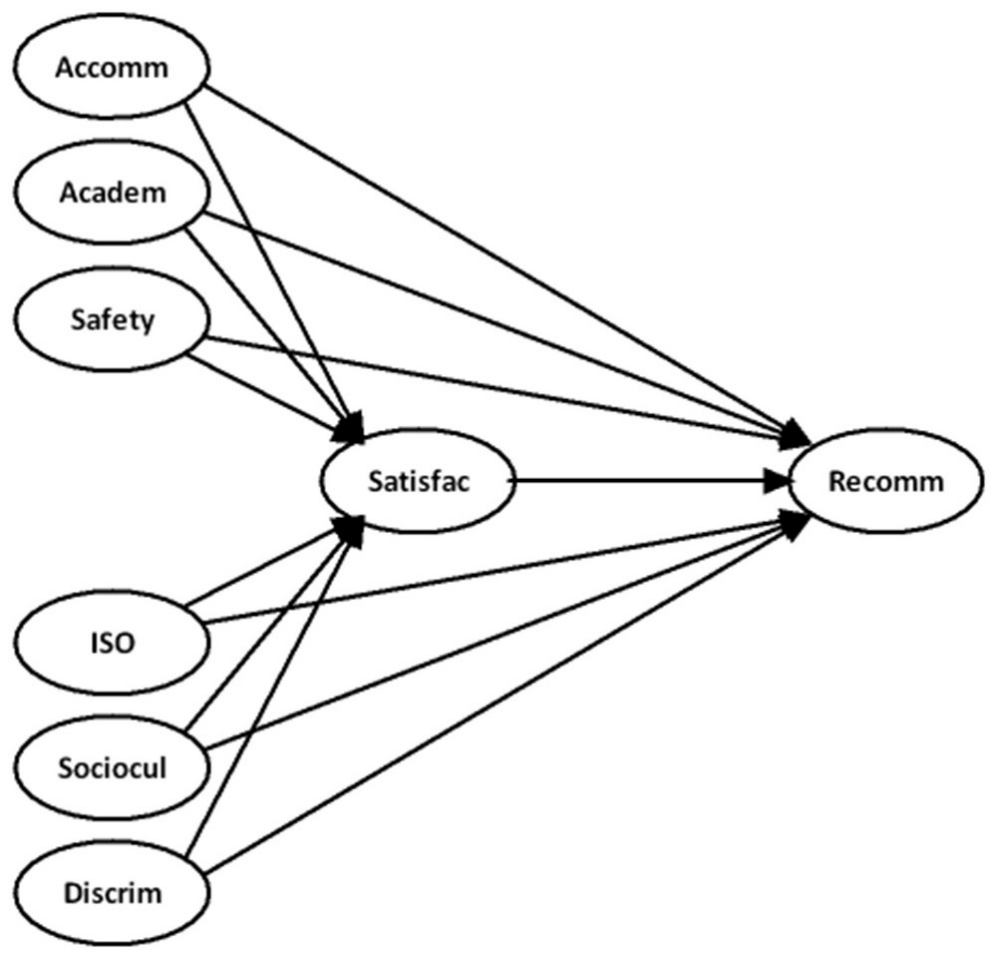
Hypothesized model.

Research shows that student satisfaction is related to the match between student priorities and the campus environment (Borden, 1995) and is being shaped continually by repeated experiences in campus life (Elliott and Shin, 2002). Prior literature on the experience of international students revealed that the overall experience of these students had a positive direct effect on their institutional recommendations (Ammigan and Jones, 2018) and satisfaction (Chelliah et al., 2019). Satisfaction has a positive and significant link with the likelihood of an institutional recommendation or an intention to revisit (Mavondo et al., 2004; Chelliah et al., 2019). The detailed theoretical explanations for the inter-construct relationships depicted in Figure 1 will be further explained in this section.

### Accommodation Experience

Living at the accommodation provided by the University may have a positive impact on the experiences of international students (Paltridge et al., 2010). In this regard, past research suggested that University accommodation may assist in creating a sense of belonging among international students (Johnson et al., 2007). Previous literature also confirmed that living on campus facilitates international students to form new social networks (Sawir et al., 2008). Besides, international students expect their accommodation not only to be available by universities but at a reasonable cost with minimum standards of comfort (Arambewela and Hall, 2009).

Life outside the classroom can be a critical aspect of the experience of any international student on campus (Ammigan, 2019). The establishment of support networks in the early stages of University life is particularly important. The quality of the accommodations is a significant factor in improving the experience of international students in a host country (Ammigan and Jones, 2018). Accordingly, the accommodations provided by the host institute should be suitable for the number of students to whom they are assigned, and they should have all of the facilities necessary for daily life, such as Wi-Fi, air conditioning, hot and cold water for daily use, and cooking facilities. With regard to the living experience of international students, Ammigan and Jones (2018) also found that the quality of the housing had the greatest influence on their satisfaction level.

### Academic Experience

International students have to adapt to a new academic environment for course selection, communication with teachers, understanding lectures, and a new assessment system (Hussain and Shen, 2019). Unfamiliarity with the new academic system (Lin and Yi, 1997) and the requirements of new skills lead international students to a variety of difficulties (Hussain and Shen, 2019). Research has confirmed the quality of teaching and good access to faculty are perceived to be key variables influencing student satisfaction (Arambewela and Hall, 2009).

The academic achievement of students is a major concern for teachers and governments (Doménech-Betoret et al., 2019). The academic experience includes the factors that meaningfully impact the overall learning experience of international students. These factors include the ability and expertise of lecturers, the quality of the lectures, the organization of courses and academic content, language education, and professional preparation (Ammigan and Jones, 2018). As a result, a teaching philosophy that incorporates these aspects is crucial for fulfilling the educational requirements of overseas students (Hellsten and Prescott, 2004). Prior research suggested that the conduct of overseas students in the lecture hall is frequently perceived as incorrect by the faculty members. Therefore, it is important for institutes to directly address and assess the types of support required in the academic environment by international students. A study conducted by Ammigan and Jones (2018) in English-speaking countries found that academic or learning satisfaction was significantly linked with the overall University experience of international students.

### Health and Safety

International students have to function not only in a dissimilar educational system but also in a different cultural context at a great distance from family and friends (Horne et al., 2018). Given this situation, the students themselves and their family members have concerns about encountering health issues in another country. Thus, safety is a major concern to international students and their families (Arambewela and Hall, 2009). Until recently, this issue has not appeared in public discussion for fear of undermining the market position of international students. After the recent deadly attacks on overseas students living in the USA, India, and Australia, the safety and security of international students became pressing concerns in the field of international education (Chelliah et al., 2019). At foreign destinations, numerous elements create an unsafe environment for international students, including a lack of private transportation, the need for casual employment, late work hours, and inadequate housing located in less-safe areas (Buchanan, 2014). Taking these factors into account, a recent study revealed that personal safety positively influences the satisfaction of international students (Chelliah et al., 2019).

### Support by the International Student Office

While the organization and structure of support services for overseas learners can differ significantly, Chinese universities generally have devoted offices that are intended to support international students in academic and non-academic matters. ISOs are a powerful entity in Chinese universities, and international students will engage with this office from the time of their admission until the completion of their degree program. The services provided by the ISO to international students, such as registration assistance, residential assistance, counseling services, cultural activities, tuition, and scholarship services, can be significant to maintain success on campus and academic satisfaction. Therefore, the support services provided by overseas student offices must be furnished to deal with the mental and emotional anxieties possibly caused by adjustment issues (Ammigan, 2019). Because of the importance of these services, the engagement of ISOs with overseas students cannot be overlooked. To understand the experiences of international students, it is worthwhile to investigate the role of the ISOs regarding their support to international students. To increase the level of satisfaction of international students, the service provider must be more aligned with the expectations of these students. Providing this support to international students can contribute directly to their level of satisfaction (Roberts and Dunworth, 2012) and institutional recommendations.

### Sociocultural Experience

Sociocultural experience is comprised of the perception of international students of how they were treated while studying abroad and the cultural and psychological problems they encountered (Wen et al., 2018). International students are a high-risk population who are vulnerable to stress due to the process of adapting to a new country (Amado et al., 2020). Prior studies such as Brisset et al. (2010) suggest that personality variables such as attachment style, trait anxiety, and extroversion may influence the ability of overseas students to make networks, and in turn influence their sociocultural and mental adaption.

Research conducted on the experience of overseas students in China exposed that these students experience difficulties with sociocultural adjustments (Wen et al., 2018). International students may wish to uphold their inherited sociocultural values and behaviors, whereas students from the host country may expect international students to integrate or assimilate their approaches to be more in line with the culture of the host country. Thus, better sociocultural settings of overseas students in the host country will have a significant impact on their level of satisfaction. Therefore, the sociocultural setting will influence the institutional recommendations of international students.

### Discrimination

Discrimination against international students varies depending on their race and nationality. Research has revealed that international students have often been stereotyped based on assumptions about their culture, linguistic backgrounds, and other differences (Siczek, 2015). Compared to domestic students, international students reported experiencing higher levels of discrimination.

Additional research revealed that discrimination affected the satisfaction of international students with their academic programs and social relations (Horne et al., 2018; Wekullo, 2019). Echoing these findings, Harrison (2010) concluded that this discrimination can be harsh, which generates noteworthy fear among overseas students. Intense discrimination may also decrease the number of international students. However, favorable perceptions of international students can lead to satisfaction with the host country as well the recommendations of their current institute to future international students.

### Institutional Recommendation

Recommendations from acquaintances, family, and friends can be key factors in the decision-making procedure of international students regarding their institutional and destination choice (Mavondo et al., 2004). This recommendation can be made orally or electronically by word of mouth. For example, to obtain information, international students may search for independent online resources written by prior students because they believe these accounts are more detailed and trustworthy compared to the testimonies delivered by universities (Gomes and Murphy, 2003). Ammigan (2019) revealed that the experiences of international students influence the recommendations of their current institutions to future students. Therefore, the experiences of international students with the academic environment of the host country, sociocultural situation, discrimination (Wekullo, 2019), accommodations, safety and security (Arambewela and Hall, 2013), and support services (Ammigan and Jones, 2018) play an important role in the institutional and destination choices of international students. Accordingly, Ammigan (2019) and Mavondo et al. (2004) found that the students who were more satisfied with their in-country experience were more expected to recommend their host University to future international students. Past research acknowledged strong relations between the quality of the experience of international students and their favorable future behavioral intentions (Boulding et al., 1993). Thus, we hypothesized that:

**H1:** A higher level of satisfaction among international students with their experience will have a greater positive impact on their institutional recommendation.
**H1a:** A higher level of satisfaction among international students with their accommodations and living arrangements will have a greater impact on their institutional recommendation.
**H1b:** A higher level of satisfaction among international students with their academic experience will have a greater impact on their institutional recommendation.
**H1c:** A higher level of satisfaction among international students with their health and safety experience will have a greater impact on their institutional recommendation.
**H1d:** A higher level of satisfaction among international students in their interactions with the ISO will have a greater impact on their institutional recommendation.
**H1e:** A higher level of satisfaction among international students with their sociocultural experience in a host country will have a greater impact on their institutional recommendation.
**H1f:** Fewer instances of discrimination against international students will have a greater impact on their institutional recommendation.

### The Satisfaction of International Students

The satisfaction of international students depends on their evaluation of the services provided by academia, such as (among other things) the level of academic and teaching services, social climate, infrastructure, and support facilities (Wiers-Jenssen et al., 2002). Elliott and Shin (2002) suggested that international student satisfaction is a dynamic process and a continually changing construct. Based on student feedback, ensuring satisfaction requires effective and clear action.

Thus, it is critical for higher education administrators and practitioners to have a comprehensive understanding of the elements that influence the experience and satisfaction of overseas students. The literature has revealed that overseas students whose educational experience exceeded their expectations were found to be more satisfied (Appleton-Knapp and Krentler, 2006). Similarly, a study showed that the dimensions of the experience of international students such as the support services, living, and learning had a significant effect on their overall satisfaction level (Ammigan and Jones, 2018). Furthermore, the satisfaction of international students influenced their institutional recommendation. Thus, we make the following hypotheses.

**H2:** Student satisfaction mediates the relationship between the experience of international students and their consequent institutional recommendation.
**H2a:** Student satisfaction mediates the relationship between the accommodation and living experience of international students and their consequent institutional recommendation.
**H2b:** Student satisfaction mediates the relationship between the academic experience of international students and their consequent institutional recommendation.
**H2c:** Student satisfaction mediates the relationship between the health and safety experience of international students and their consequent institutional recommendation.
**H2d:** Student satisfaction mediates the relationship between the experience of students with the ISO and their consequent institutional recommendation.
**H2e:** Student satisfaction mediates the relationship between the sociocultural experience of international students and their consequent institutional recommendation.
**H2f:** Student satisfaction mediates the relationship between the non-discrimination experience of international students and their consequent institutional recommendation.

## Methodology

A structured questionnaire comprised of questions with lists of precoded items was used to collect the data for analyzing the experience of international students who are studying in China. The questionnaire was sent to students who have at least 6 months of living and studying experience in China, and the objective of the data collection was also explained to all of the participants. A pretest was conducted (*n* = 30) to ensure the appropriateness of the wording of the questionnaire, and statistical criteria were used in the study.

The data were collected in the three Chinese Universities (Sichuan, SWUFE, and UESTC) located in Chengdu city. A total of 302 international students responded to the survey questionnaire. All of the data were collected through www.wjx.cn. After eliminating the incomplete questionnaires (*n* = 24), a final sample of 278 respondents who lived and studied in China was collected for further processing. Roughly 30.94% of the respondents were from Africa, 42.80% were from Asia, and 26.26% were from developed regions. A majority of the participants were enrolled in Master (34.17%) or PhD programs (31.65%), which enhances the heterogeneity of the sample pool. In Table 1, a detailed overview of the demographic sample is presented.

**Table 1:** Demographics of respondents.

		**Developing world vs. Developed world**				
		**Asia**	**Africa**	**Australia**	**Europe**	**USA**
	**Categories**	**Frequency**	**Frequency**	**Frequency**	**Frequency**	**Frequency**
Gender	Male	103	57	2	23	7
	Female	16	29	3	31	7
Program enrolled	Bachelor's	24	30	1	20	7
	Master's	41	27	3	20	4
	PhD	54	29	1	2	2
	Chinese diploma/exchange				12	

All of the items were measured using a 5-point Likert scale with response choices ranging from 1 = strongly disagree to 5 = strongly agree. In line with other empirical studies, the measurement items based on previous research in the context of the experience of international students were modified to better fit the context of this study. Four items were used to calculate the accommodation experiences of international students. Among these items, three were adopted from Santos (2018) and one came from Ammigan (2019). Three items were used to measure the overall learning experience of students (Ammigan, 2019). The health and safety variable were measured using three items from Chelliah et al. (2019). Questions regarding the support services provided by the ISO were taken from Chelliah et al. (2019). The perceived discrimination experience was measured using two items from Wekullo (2019) and one item from Harrison (2010). Three items were used to gauge the satisfaction of international students, and they were adopted from Chelliah et al. (2019). One item for the institutional recommendation was adapted from Mavondo et al. (2004) and two others came from Chelliah et al. (2019). For a detailed list of these items, please see the Appendix.

## Results

### Descriptive Statistics

The means, SDs, and correlations for all of the variables are presented in Table 2. To check the internal consistency and reliability of the studied variables, we measured Cronbach's alphas and found that the values of all of the variables are higher than the recommended level of 0.70 (Fornell and Larcker, 1981).

**Table 2:** Descriptive statistics and correlations.

	**1**	**2**	**3**	**4**	**5**	**6**	**7**	**8**	**9**	**10**	**11**
Gender	–										
Education	−0.227^**^	–									
Country	0.237^**^	0.004	–								
Accommodation	−0.103	0.049	−0.001	(0.772)							
Academic	−0.079	0.020	−0.003	0.456^**^	(0.722)						
Safety	−0.135^*^	0.058	0.042	0.424^**^	0.427^**^	(0.713)					
ISO	−0.035	0.019	0.010	0.386^**^	0.479^**^	0.395^**^	(0.888)				
Social	−0.019	−0.010	0.029	0.282^**^	0.341^**^	0.427^**^	0.271^**^	(0.725)			
Discrimination	−0.015	0.040	0.286^**^	0.311^**^	0.340^**^	0.300^**^	0.289^**^	0.349^**^	(0.763)		
Satisfaction	−0.048	0.079	0.122^*^	0.601^**^	0.589^**^	0.473^**^	0.530^**^	0.434^**^	0.430^**^	(0.842)	
Recommendation	−0.016	0.111	0.100	0.516^**^	0.506^**^	0.459^**^	0.606^**^	0.329^**^	0.420^**^	0.808^**^	(0.943)
Mean	1.31	2.10	2.36	3.705	3.558	3.998	3.371	3.741	3.524	3.718	3.513
Std. deviation	0.462	0.891	1.435	0.8450	0.9025	0.7464	1.099	0.7247	0.9596	0.9304	1.137

* *p < 0.05*,

** *p < 0.01*.

*The off-diagonal numbers in parentheses are the values of Cronbach's alpha*.

The validity of the construct was tested by both discriminant and convergent. By performing Exploratory Factor Analysis (EFA), both discriminant and convergent validity were confirmed (Avsec and Jamšek, 2018). Second, to verify both the concurrent and predictive validity, Pearson's corrected coefficient r_xy_ was examined (Odom and Morrow, 2006). Correlation between individual items was all found below the threshold level of r_xy_ < 0.7 (Miller et al., 2009; Rossiter, 2011). The output of validity measurement thus revealed that all test items are appropriately constructed and designed to measure what they supposed (Avsec and Jamšek, 2018). Therefore, we avoided overlapping test items.

### Hierarchical Regression

To test the hypotheses, a series of hierarchical regression analyses were conducted. First, control variables such as gender, education, and country were entered into the regression model. Then, the experiences of international students such as accommodations, academics, health and safety, support services by the ISO, sociocultural factors, and discrimination were entered into the model.

After controlling the effects of the demographic variables, as shown in Table 3, we added the experiences of international students (the accommodation, academics, health and safety, support services by the ISO, socioculture, and discrimination) in M2. With this addition, the explanatory power of the regression model was significantly improved (Δ*R*^2^ = 0.512, *p* < 0.01). Consequently, Hypothesis 1 was supported partially, which means that the accommodation [*β* = 0.217 (*t* = 4.345), *p* < 0.00], academics [β = 0.126 (*t* = 2.403), *p* < 0.05], health and safety [β = 0.114 (*t* = 2.206), *p* < 0.05], support services by the ISO [β = 0.367 (*t* = 7.372), *p* < 0.00], and discrimination [β = 0.147 (*t* = 2.976), *p* < 0.00] had a significant positive impact on the recommendations of the current institute to future students. Hence, Hypotheses 1a, 1b, 1c, 1d, and 1f were supported. However, 1e was rejected, as the socioculture experience of international students was shown to have a non-significant impact on the institutional recommendation [β = 0.026 (*t* = 0.535), ns].

**Table 3:** Regression results.

	**Institutional recommendation**			**Student satisfaction**	
**Variables**	**M1**	**M2**	**M3**	**M4**	**M5**
**Demographics**					
Gender	−0.016	0.060	0.051	−0.066	0.015
Education	0.107	0.092^*^	0.058	0.063	0.052
Country	0.103	0.035	−0.020	0.138	0.083
**Students' experience**					
Accommodation		0.217^**^	0.012		0.315^**^
Academics		0.126^*^	−0.031		0.241^**^
Safety		0.114^*^	0.075		0.060
ISO		0.367^**^	0.237^**^		0.200^**^
Socioculture		0.026	−0.070		0.148^**^
Discrimination		0.147^**^	0.084^*^		0.097^*^
**Mediator**					
Satisfaction			0.653^**^		
*R* ^2^	0.022	0.534	0.711	0.025	0.585
Δ*R*^2^	0.022	0.512^**^	0.177^**^	0.025	0.560^**^

* *p < 0.05*,

** *p < 0.01*.

Then, we followed the procedure proposed by Kenny and Baron (1986) to test the mediation effect. Accordingly, we entered satisfaction into the model in M3, and we found that the explanatory power with respect to the institutional recommendation was improved (Δ*R*^2^ = 0.177, *p* < 0.01).

As shown in Table 3, the satisfaction of students was positively related to their institutional recommendation (β = 0.653, *p* < 0.01). The accommodation [β = 0.012 (*t* = 0.271), ns], academic experience [β = −0.031 (*t* = −0.723), ns], health and safety [β = 0.075 (*t* = 1.824), ns], and support services by the ISO [β = −0.070 (*t* = −1.812), ns] all had a non-significant impact on the recommendations of students of their current institute to future students. However, the impacts of support services by the ISO [β = 0.237 (*t* = 5.832), *p* < 0.00] and discrimination [β = 0.084 (*t* = 2.131), *p* < 0.05] on the institutional recommendation were still significant (β = 0.125, *p* < 0.05). As shown in M5, after controlling the effects of the demographic variables, adequate accommodation [β = 0.315 (*t* = 6.673), *p* < 0.00], academic experience [β = 0.241 (*t* = 4.864), *p* < 0.00], support services by the ISO [β = 0.200 (*t* = 4.249), *p* < 0.00], socioculture [β = 0.148 (*t* = 3.231), *p* < 0.00], and discrimination [β = 0.097 (*t* = 2.079), *p* < 0.05] had a significant positive impact on the satisfaction of students, while health and safety had a non-significant impact on their satisfaction [β = 0.060 (*t* = 1.237), ns].

In conclusion, Hypothesis 2 was partially supported: H2a, H2b, H2d, H2e, and H2f were supported, whereas H2c was rejected.

### Supplementary Analysis

**Figure 2:**
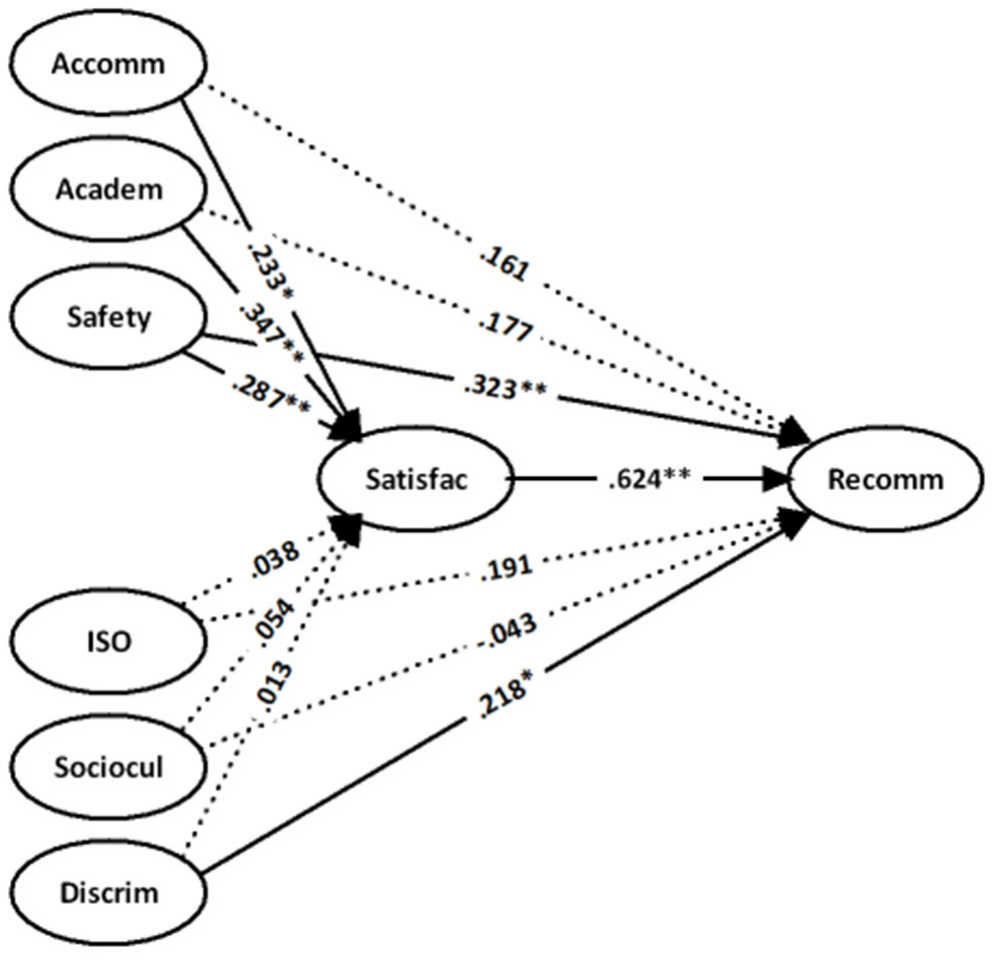
Results for the African (country code = 1) sample with controls for gender and education.

**Figure 3:**
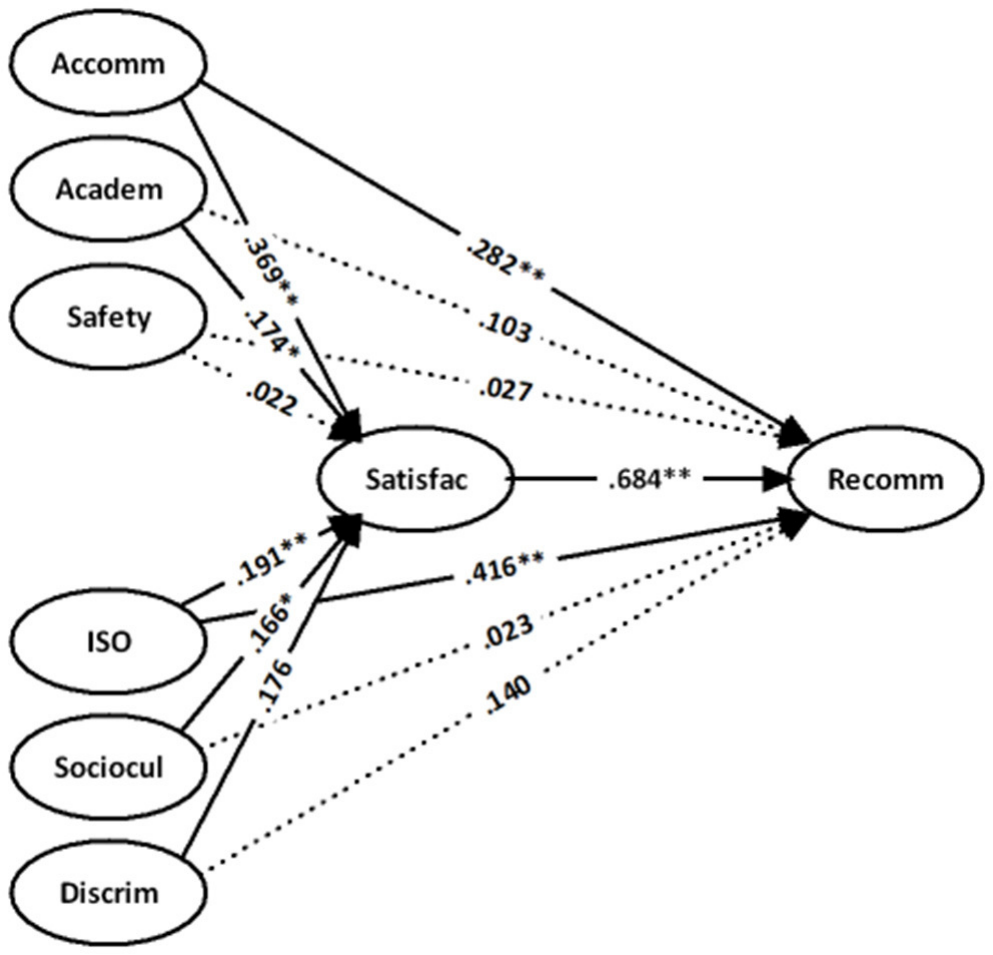
Results for the Asian (country code = 2) sample with controls for gender and education.

**Figure 4:**
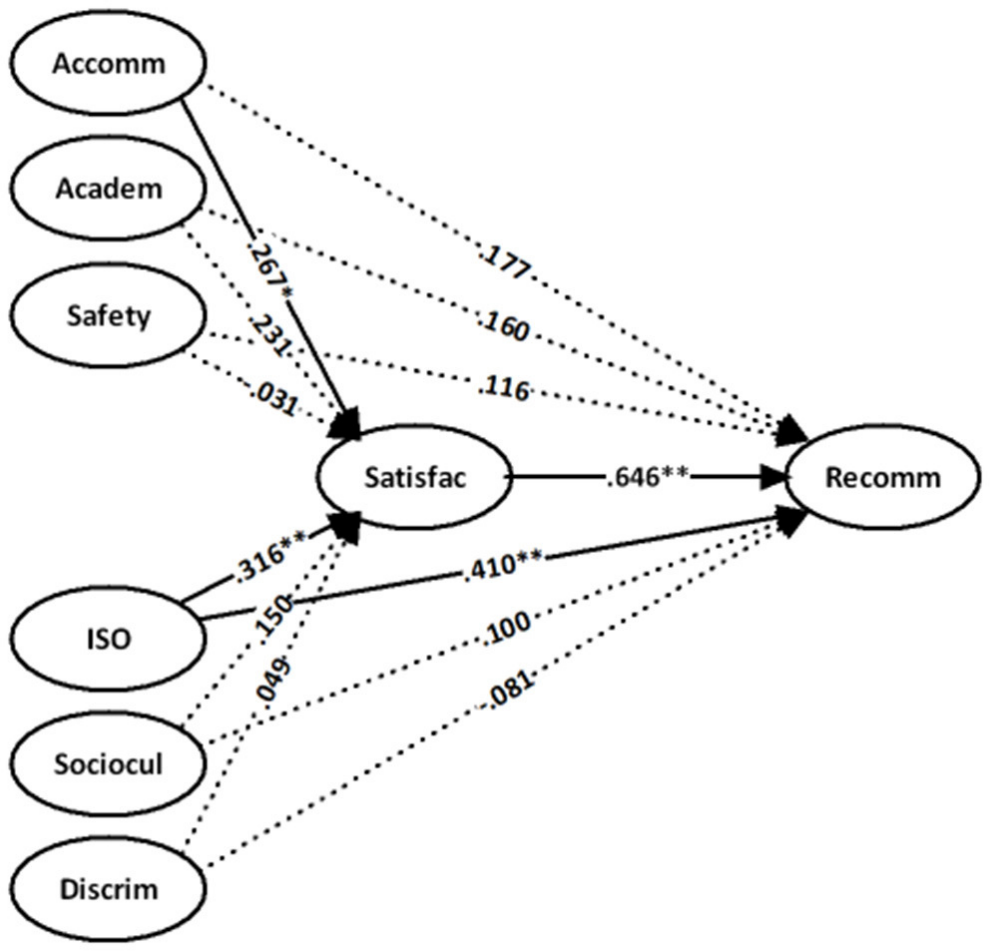
Sample from developed regions [country code = (3+4+5) 3] with controls for gender and education.

We designed a supplementary analysis to further investigate whether there are differences between the perceived experiences of students from different regions in the above models. First, we divided the sample into three subsamples according to the developing (Africa and Asia excluding South Korea, Singapore, and Japan) and developed (America, Australia, and Europe) regions. After the above steps, the regression analysis was undertaken. The difference between this analysis and the previous analyses is that country is no longer a control variable. The results are shown in Tables 4–6, Figures 2–4.

**Table 4:** Results of African students.

	**Institutional recommendation**			**Student satisfaction**	
**Variables**	**M1**	**M2**	**M3**	**M4**	**M5**
**Demographics**					
Gender	−0.103	0.110	0.101	−0.164	0.016
Education	0.167	0.212^*^	0.131	0.074	0.131
**Students' experience**					
Accommodation		0.161	0.015		0.233^*^
Academics		0.177	−0.040		0.347^**^
Safety		0.323^**^	0.144		0.287^**^
ISO		0.191	0.168^*^		0.038
Socioculture		−0.043	−0.077		0.054
Discrimination		0.218^*^	0.209^**^		0.013
**Mediator**					
Satisfaction			0.624^**^		
*R* ^2^	0.051	0.507	0.691	0.041	0.527
Δ*R*^2^	0.051	0.457^**^	0.184^**^	0.041	0.486^**^

* *p < 0.05*,

** *p < 0.01*.

**Table 5:** Results of Asian students.

	**Institutional recommendation**			**Student satisfaction**	
**Variables**	**M1**	**M2**	**M3**	**M4**	**M5**
**Demographics**					
Gender	−0.126	−0.032	−0.022	−0.129	−0.015
Education	0.141	0.091	0.007	0.171	0.122^*^
**Students' experience**					
Accommodation		0.282^**^	0.029		0.369^**^
Academics		0.103	−0.016		0.174^*^
Safety		0.027	0.012		0.022
ISO		0.416^**^	0.285^**^		0.191^**^
Socioculture		0.023	−0.091		0.166^*^
Discrimination		0.140	0.019		0.176^*^
**Mediator**					
Satisfaction			0.684^**^		
*R* ^2^	0.039	0.603	0.748	0.050	0.690
Δ*R*^2^	0.039	0.563^**^	0.145^**^	0.050	0.640^**^

* *p < 0.05*,

** *p < 0.01*.

**Table 6:** Results of the respondents from developed regions.

	**Institutional recommendation**			**Student satisfaction**	
**Variables**	**M1**	**M2**	**M3**	**M4**	**M5**
**Demographics**					
Gender	0.259^*^	0.172^*^	0.127	0.161	0.069
Education	0.012	0.009	0.062	−0.091	−0.082
**Students' experience**					
Accommodation		0.177	0.005		0.267^*^
Academics		0.160	0.011		0.231
Safety		0.116	0.136		−0.031
ISO		0.410^**^	0.205^*^		0.316^**^
Socioculture		0.100	0.003		0.150
Discrimination		−0.081	−0.113		0.049
**Mediator**					
Satisfaction			0.646^**^		
*R* ^2^	0.067	0.559	0.735	0.038	0.580
Δ*R*^2^	0.067	0.493^**^	0.175^**^	0.038	0.542^**^

* *p < 0.05*,

** *p < 0.01*.

In general, for the students from both developing and developed regions, the mechanism of experience that affects the institutional recommendation is the same. That is, the perceived experience of international students influences the recommendation of their current University to others through the mediated role of the satisfaction of students. In terms of the specific differences as per the experiences of the samples of African students shown in Table 4 and Figure 2, academic experiences [β = 0.437 (*t* = 3.739), *p* < 0.01] and health and safety [β = 0.287 (*t* = 2.721), *p* < 0.01] were the strongest factors in determining the satisfaction and institutional recommendation of students. These factors were followed by the accommodation [β = 0.233 (*t* = 2.575), *p* < 0.05]. However, discrimination [β = 0.218 (*t* = 2.604), *p* < 0.05] and ISO services [β = 0.168 (*t* = 2.174), *p* < 0.05] were revealed to have a direct impact on the recommendation. More importantly, a significant direct relationship between the ISO and the institutional recommendation was identified after we entered the satisfaction of students as a mediating variable. Conversely, non-significant direct (between the socioculture and the satisfaction) and indirect (between the socioculture and the recommendation) relationships revealed that African students in China face more problems in understanding and adapting to the local sociocultural values.

As shown in Table 5 and Figure 3, Asian students give more weight to the factors of the accommodation and the ISO as the strongest predictors of the satisfaction of students and the recommendation of their current institute to others. These two factors are followed by academic, sociocultural, and discrimination experiences. In addition to the impact on the satisfaction of students, the accommodation and ISO can also directly affect the institutional recommendation. However, non-significant direct [health and safety and recommendation; β = 0.027 (*t* = 0.348), ns] and indirect [between health and safety and satisfaction; β = 0.022 (*t* = 0.324), ns] relationships revealed that Asian students in China face troublesome health and safety issues.

As shown in Table 6 and Figure 4, students from developed countries who are more satisfied with the services provided by the ISO [β = 0.316 (*t* = 2.896), *p* < 0.01] are more likely to recommend their current institution to future students. This factor is followed by the accommodation services [β = 0.267 (*t* = 2.634), *p* < 0.05]. However, the academic, health and safety, discrimination, and sociocultural dimensions of the experience of international students from developed countries revealed no direct influence on the institutional recommendation and indirect influence through the satisfaction of students.

## Discussion

The decision to recruit international students has been considered as both a national strategy to strengthen the international competitiveness and soft power of China and a diplomatic strategy to reposition Chinese higher education in the world. To reap the full benefits of hosting international students and sustain the current growth in the international student market, it is key to examine the experience of international students as an issue of customer satisfaction and understand the factors that influence the recommendations of their current institute to future students. Accordingly, this study performed an analysis of the experience of international students with their accommodations, academic opportunities, health and safety services, services by the ISO, sociocultural experiences, and discrimination to reveal the influence of these factors on their overall satisfaction and their recommendations to future students. Furthermore, past research revealed that both educational and non-educational experience varies among nationality groups (Arambewela and Hall, 2009). Accordingly, a comparative analysis has also been conducted to better understand the exact picture of the experience of international students in the host country. Based on the responses of students, the dimensions of international student experiences are discussed in the following sections.

In the main model, accommodation was revealed to not only have a positive direct effect on the institutional recommendations of students but also have an indirect positive effect through student satisfaction. In the Asian student sample, for example, positive experiences with accommodations directly influenced the recommendations of students. In addition, these experiences influenced the overall satisfaction of students, which in turn indirectly influenced their recommendations. Notably, African students and students from developed regions were shown to have an indirect relationship through satisfaction. These outcomes revealed that international students, in general, are willing to recommend their current institute to future international students based on their accommodation experience. In the context of student regions, Asian students were revealed to have the strongest positive effect on both the satisfaction and the recommendation, followed by African students and students from developed regions. Thus, students from developed (0.267, *p* < 0.05) regions and Africa (0.233, *p* < 0.05) are relatively less satisfied with their experience in China. This difference could be because the quality of the dormitories in Chinese universities was not up to western standards (Ping et al., 2019), because the dormitory buildings for international students are often far from central areas of University activity (Ding, 2016), or because classrooms and dormitories are located on different campuses. To improve the accommodation experiences of students from developed regions and Africa, institutions must review their accommodation facilities to determine whether all of the necessary amenities have been provided. This review would include ensuring that the environment of dormitories is suitable for studying and the dormitories are situated not far from school.

Academic experience has a positive direct effect on the institutional recommendation in the main model. Moreover, in common with the accommodations, academic experience also has an indirect effect on student satisfaction. In the supplementary analysis, African students were revealed to have a stronger (0.347, *p* < 0.01) relationship between academic experience and overall satisfaction. In contrast to African students, a relatively weak relationship was found between the academic experiences of Asian students and the level of their satisfaction (0.174, *p* < 0.05). Moreover, students from developed regions displayed a non-significant direct relationship between the academics and their recommendations or an indirect effect through their satisfaction. These results reveal that the students from Africa appear to be satisfied with the academic experience in China, while Asian students may be relatively less satisfied. However, students from developed regions are neither satisfied nor willing to recommend their current institute to future international students based on their academic experience. These results are consistent with a prior study in which Ding (2016) found low levels of satisfaction among international students with their study experience in Chinese universities.

Major challenges for international students include inadequate student-faculty interactions on campus (Wen et al., 2018), a lack of teachers with adequate English skills, and misunderstandings of teaching methodologies. There may also exist cultural differences in the relationship between teacher behaviors and the learning of students (Jiang et al., 2021). Therefore, instructors need to better understand the cultures of international students, including their approaches to both learning and teaching, by familiarizing themselves with the prior learning experiences of these students. Further, while considering the diversity of international students and to cater to the specific pedagogical demands of students from all around the globe, academia will need to adapt non-traditional teaching techniques.

To fulfill the learning requirements of international students and to meet the quality of universities from developed regions, institutions must recruit qualified foreign and local teaching and management staff in the various areas of study. Accordingly, the Chinese government should encourage institutions to hire foreign teachers, and policies should be introduced to attract and retain foreign talent. Moreover, to establish intercultural awareness within departments, institutions should provide intercultural training to both Chinese faculty and international students.

Health and safety were revealed to only have a positive direct effect on the institutional recommendation in the main model, while the indirect effect through satisfaction has a non-significant effect. For the African student sample, health and safety factors influenced the recommendations directly. Health and safety factors also influenced the recommendations indirectly through the overall satisfaction. These outcomes revealed that African students, in general, are satisfied with their health and safety experience in China. Furthermore, African students are willing to recommend their current institute to future international students based on their health and safety experience. However, a non-significant direct relationship was found between health and safety and the institutional recommendation, and an indirect relationship through satisfaction among the students from developed regions and Asia was uncovered. These relationships imply that these students have concerns regarding their health and safety in China. It is important to question why African students are more satisfied with health and safety compared to their Asian and Western counterparts. It may be that the provided health benefits and overall safety in China are preferable to their home countries. However, the students from developed regions and Asia have different perceptions regarding the provided health benefits and the safety measures in China. In most European countries, for instance, free medical facilities are provided. To deal with the health and safety issues of international students, institutions should inform incoming students about the health facilities available for them. Institutions should also guide if international students have to purchase health insurance, and they should clearly explain the extent of this health insurance coverage. For the safety of students, new international students should be guided through the campus or dormitory environment and given introductions to other important local customs, norms, rules, and regulations.

In the main model, services provided by the ISO have a positive direct effect on the institutional recommendation and an indirect effect through student satisfaction. Among Asian students and students from developed regions, the ISO services have both direct influence on the recommendation and indirect influence through satisfaction. These results demonstrate that international students can use their own positive experiences with the ISO as an influencing factor to recommend their current institution to future students. Notably, the experience of African students only revealed a direct relationship between the ISO and the institutional recommendation. Thus, African students could have concerns with the ISO staff or their policies. Therefore, representatives of the ISO should listen to the issues faced specifically by African students.

With respect to the international discrimination experience in China in the main model, discrimination was found to have a positive direct effect on the institutional recommendation and an indirect effect through student satisfaction. In the supplementary analysis, a direct positive relationship between the discrimination experience and the institutional recommendation (0.218, *p* < 0.05) was confirmed by the experiences of African students. This relationship was the strongest (0.209, *p* < 0.01) after we added the student satisfaction into the model. However, Asian students revealed a relatively low-level (0.176, *p* < 0.05) relationship between the perceived discrimination and their satisfaction. This finding appears to show that African students are fully satisfied with their experience, and Asian students also seem to be satisfied to some extent with the perceived non-discrimination experience in China. Based on their non-discriminatory experience, these students are willing to recommend their current institute to future international students.

Remarkably, a non-significant direct relationship between the perceived discrimination and the recommendation, or an indirect relationship *via* the student satisfaction, was revealed among the students from developed regions. It should also be noted that students from developed regions do not seem to be satisfied with their sociocultural experience in China. Consequently, due to differing cultural and social backgrounds, the attitudes of local people and faculty members could be perceived as discriminatory by students from developed regions. Asian strategies of managing the classroom environment and the expectations of a teacher or supervisor from international students could affect the perception of discrimination of Western students. In China, students tend to be obedient in classes (Li and Wegerif, 2014) and classes are more teacher-centered (Haley and Ferro, 2011). In the Western educational philosophy, however, the relationship between the student and the teacher is considered mutual (Qi, 1999). Therefore, the unfamiliarity of students with Asian customs and formalities could be one reason why their discrimination perception does not positively influence their recommendation.

In this regard, researchers have suggested ongoing professional development activities focusing on how to respond to linguistically and culturally varied students (Siczek, 2015). Lee and Rice (2007) suggested that creating campus events would help incorporate these students and create awareness of the challenging environment faced by the international students. Moreover, international students not only need to be informed of local traditions, but they also need to be familiar with the roles and statuses of teachers in Asian society.

In the main model, the sociocultural experience was discovered to have only an indirect effect through the student satisfaction on the recommendation. For Asian students, in particular, satisfaction with their sociocultural experience in China influenced their recommendations to future students. However, no direct (between the sociocultural experience and the recommendation) or indirect (through satisfaction) relationship was revealed among the students from developed regions and Africa. These outcomes demonstrated that international students from Africa and developed regions face difficulties understanding Chinese culture and risk cultural misunderstandings with local peoples. Prior research also indicated that major challenges for international students in China include integrating with local students (Ma and Wen, 2018) and the difficulties in sociocultural adjustment (Wen et al., 2018).

Therefore, international students in general and those from African and developed countries, in particular, do not appear to be satisfied with their sociocultural experience in China. Consequently, they are unwilling to recommend their current institute to future international students based on this experience. However, Asian students are more willing to recommend their current institute to future students due to their sociocultural experiences. This outcome may be because the cultural background of these students makes it easy for them to understand local norms and customs, and as a result, they do not feel as much discomfort in Chinese culture compared to their African and Western counterparts. To improve the sociocultural experiences of international students, universities and local governments can arrange cultural activities (such as short live dramas or movies) that display local customs and norms. Similarly, regular meetings, sports events, food galas, and other such activities between local and international students can also increase the understanding of newcomers of local traditions.

## Conclusion

In an increasingly competitive global market of international education, the host country must continue concentrating on the overall experience of international students in regard to increasing their satisfaction rating and subsequent recommendations of their institutions to others. Consequently, to advance the overall quality of the services provided to overseas students and to maintain sustainable growth in the market of international students, aggressive policies have been formulated by the governments of other major study destinations for international students (Ding, 2016). However, most of the related research has been conducted in native English-speaking countries. Therefore, the current literature lacks sufficient research that provides an inclusive understanding of the individual experiences, expectations, and motivations of students in non-English speaking countries (Calikoglu, 2018). As a result, the objective of this research is to examine the experiences of international students in China.

The outcomes of this study revealed that establishing an ISO for the sake of assisting international students with University registration, residence help, counseling services, personal issues, trips, tuition fees, scholarships, and arrangements of cultural activities in a non-English speaking country is seen as an influential and encouraging step by international students. Contrary to the previous studies on international students in China (e.g., Ding, 2016), this study reveals a slight improvement in the accommodation facilities in Chinese universities. However, the findings of the current research confirmed the overall experience of international students in China, including the academic and other experiences, to be below the international benchmark. Participants from developed regions only appear to be satisfied with the ISO and the accommodations, and they are willing to recommend their current institute to future students only based on their experience with these elements. Additionally, the outcomes of the current research exposed that regardless of their origin (in developing or developed regions), international students face inconveniences regarding their sociocultural and health, and safety experiences. Consequently, this study recommends that to compete with other studies abroad destinations and to improve the experiences of international students, China should formulate comprehensive policies to support international students.

## Data Availability Statement

The raw data supporting the conclusions of this article will be made available by the authors, without undue reservation.

## Author Contributions

MJ developed the conceptual notions and drafted the manuscript. TQ and AM contributed in literature, methods, and analysis. MW and SZ reviewed the manuscript critically, provided substantial contributions, and approved the final version to be submitted. All authors contributed to the article and approved the submitted version.

## Conflict of Interest

The authors declare that the research was conducted in the absence of any commercial or financial relationships that could be construed as a potential conflict of interest.

## Publisher's Note

All claims expressed in this article are solely those of the authors and do not necessarily represent those of their affiliated organizations, or those of the publisher, the editors and the reviewers. Any product that may be evaluated in this article, or claim that may be made by its manufacturer, is not guaranteed or endorsed by the publisher.
