# Supplementary material for: The Experience of International Students and Institutional Recommendations: A Comparison Between the Students From the Developing and Developed Regions
Source: Front Psychol. 2021 Aug 13;12:667230. doi: 10.3389/fpsyg.2021.667230 (PMC8415018; doi:10.3389/fpsyg.2021.667230)
Supplement: Supplementary file 1 [file Presentation_1.pdf]

**Appendix (Questionnaire's items)**

Accommodation experience (Santos, 2018; Ammigan, 2019)

1) All of the necessary facilities have been provided at my accommodation

2) The location of my accommodation is convenient for going to school

3) A good environment is provided at my accommodation

4) Access to suitable accommodations has been given

Academic experience (Ammigan, 2019)

4) The academic staff have a good command of English

5) Lecturers are experts

6) I have easy access to the academic staff

Health and Safety experience (Chelliah et al. 2019)

1) A healthy and safe environment is provided by the university

2) Student health issues are responded to in a timely fashion

3) In general, there is a safe environment for international students inside or outside of campus

Support services experience (Chelliah et al. 2019)

1) The staffs from the international student office listen to the students' concerns

2) The staffs from the international student office perform their duties in friendly manner

3) The staffs from the international student office are always willing to help a student to ease his or her discomfort

Sociocultural experience (Wen et al., 2018)

1) I have become accustomed to the pace of life in China

2) I understand the Chinese value system

3) I have adapted to the local etiquette

4) It is easy for me to make local friends

Discrimination experience (Wekullo, 2019; Harrison, 2010)

1) I never face a discriminatory attitude from faculty
2) I never face a discriminatory attitude from classmates or other students
3) I never face a discriminatory attitude from local people

Student satisfaction (Chelliah et al. 2019)

1) Based on all of the experiences, I feel that attending this university was the right decision
2) I am happy with the learning support given by the lecturers
3) In general, I am satisfied with the facilities

Recommendation to others (Chelliah et al. 2019; Mavondo, et al., 2004)

1) I will recommend my university to other international students for their future studies
2) This university will be the first choice when I need to recommend an institution to others
3) Based on my satisfaction with the university, I am more likely to recommend it to future students
